# Discovery of Potential Inhibitors for RNA-Dependent RNA Polymerase of Norovirus: Virtual Screening, and Molecular Dynamics

**DOI:** 10.3390/ijms22010171

**Published:** 2020-12-26

**Authors:** Oluwakemi Ebenezer, Maryam A. Jordaan, Nkululeko Damoyi, Michael Shapi

**Affiliations:** Department of Chemistry, Faculty of Natural Science, Mangosuthu University of Technology, 511 Mangosuthu Highway, Durban 4000, South Africa; re.korede@gmail.com (O.E.); nedamoyi@gmail.com (N.D.); mshapi@mut.ac.za (M.S.)

**Keywords:** CMX521, human norovirus, molecular similarity, virtual screening, molecular dynamics

## Abstract

Noroviruses are non-enveloped viruses with a positive-sense single-stranded RNA (ssRNA) genome belonging to the genus *Norovirus*, from the family *Caliciviridae,* which are accountable for acute gastroenteritis in humans. The *Norovirus* genus is subdivided into seven genogroups, i.e., (GI-GVII); among these, the genogroup II and genotype 4 (GII.4) strains caused global outbreaks of human norovirus (HuNov) disease. The viral genome comprises three open reading frames (ORFs). ORF1 encodes the nonstructural polyprotein that is cleaved into six nonstructural proteins, which include 3C-like cysteine protease (3CLpro) and a viral RNA-dependent RNA polymerase. ORF2 and ORF3 encode the proteins VP1 and VP2. The RNA-dependent RNA polymerase (RdRp) from noroviruses is one of the multipurpose *enzymes* of RNA *viruses* vital for replicating and transcribing the viral genome, making the virally encoded enzyme one of the critical targets for the development of novel anti-norovirus agents. In the quest for a new antiviral agent that could combat HuNov, high throughput virtual screening (HTVS), combined with e-pharmacophore screening, was applied to screen compounds from the PubChem database. CMX521 molecule was selected as a prototype for a similarity search in the PubChem online database. Molecular dynamics simulations were employed to identify different compounds that may inhibit HuNov. The results predicted that compound CID-57930781 and CID-44396095 formed stable complexes with MNV-RdRp within 50 ns; hence, they may signify as promising human norovirus inhibitors.

## 1. Introduction

The adoption of a brisk and cost-effective methodology in the discovery of new drug leads has caused pharmaceutical companies to re-evaluate R&D strategies. In the interim, a computer-aided drug design (CADD) approach, which uses substantial computational power, has become one of the more efficient searches for new lead compounds [1]. This approach includes identifying hit compounds using structure or ligand-based virtual screening and in silico simulations, chemical and biological information about ligands and molecular targets of feasible hits. The coupling of these approaches will make it easier to eliminate compounds with properties that are out of optimal ranges and select promising candidates for optimization [2,3]. The ligand-based virtual screening methods using molecular similarity searching are relatively inexpensive and widely used to pool promising molecules with comparable structures, with the probability of similar biological properties, from chemical structure databases. 

The search for anti-norovirus compounds has always remained a priority for therapeutic chemists. Different approaches have been utilized to search for potential antiviral molecules to combat the virus [4,5,6,7]. Noroviruses are non-enveloped viruses with a positive-sense single-stranded RNA (ssRNA) genome belonging to the genus *Norovirus*, family *Caliciviridae.* The *Norovirus* genus is grouped into seven genogroups (GI-GVII), of which the genogroup II and genotype 4 (GII.4) strains are known to trigger global outbreaks of HuNov disease. The viral genome comprises three open reading frames (ORFs). ORF1 encodes the nonstructural polyproteins that are cleaved into six nonstructural proteins (p48 (NS1/2), NTPase (NS3), p22 (NS4), VPg (NS5) [7,8], along with the virus-encoded 3C-like cysteine protease (3CLpro) and a viral RNA-dependent RNA polymerase (RdRp). Meanwhile, VP1 and VP2 proteins are encoded in ORF2 and ORF3, respectively [8].

Furthermore, the human norovirus (HuNov) is responsible for acute viral gastroenteritis infection, commonly known as “winter vomiting disease” [9]. Norovirus infection is not limited to any age group or setting. It can target some high-risk groups, particularly children under the age of five and the elderly, travelers, soldiers, and patients who exhibit immunodeficiency or have received organ transplantation [10,11,12,13]. In the US alone, HuNov accounts for ~23 million gastroenteritis cases annually, resulting in ~109 thousand hospitalizations and nearly 900 deaths [14]. Globally, norovirus causes ~ 685 million cases of acute gastroenteritis, and ~200 million cases are among children under the age of five, leading up to ~50 thousand child deaths every year, predominantly in developing countries [14]. Noroviruses are a sporadic epidemic and extremely contagious, and humans are the only known reservoir, thus making HuNoV infection the principal cause of death among people with viral gastroenteritis. The person-to-person contact via the fecal-oral route boosts the spread of the outbreak. The alternative mode of transmission includes the ingestion of aerosolized vomitus or indirectly via contact with contaminated environmental surfaces (i.e., through hand/mouth contact) and infected food handlers [15,16]. Low-level transmission can occur via tainted drinking supplies, when surface or groundwater supplies are contaminated, and directly infect the filter feeders, such as oysters living in contaminated waters, which become infected food sources [17,18,19]. The clinical symptoms such as queasiness, fever, and diarrhea have been reported to occur for one to three days; meanwhile, norovirus defecation lingers up to 14 days, posing a challenge in healthy adults [20,21,22,23]. Viral shedding occurs in children from 22 days to 47 days after the onset of symptoms; however, in the aged, excretion of norovirus in stools continued for 44.5 days [20,24]. There is a strong indication showing the imperative need for medical intervention, but up to date, no specific approved therapies are available to combat human norovirus infection. 

**Figure 1:**
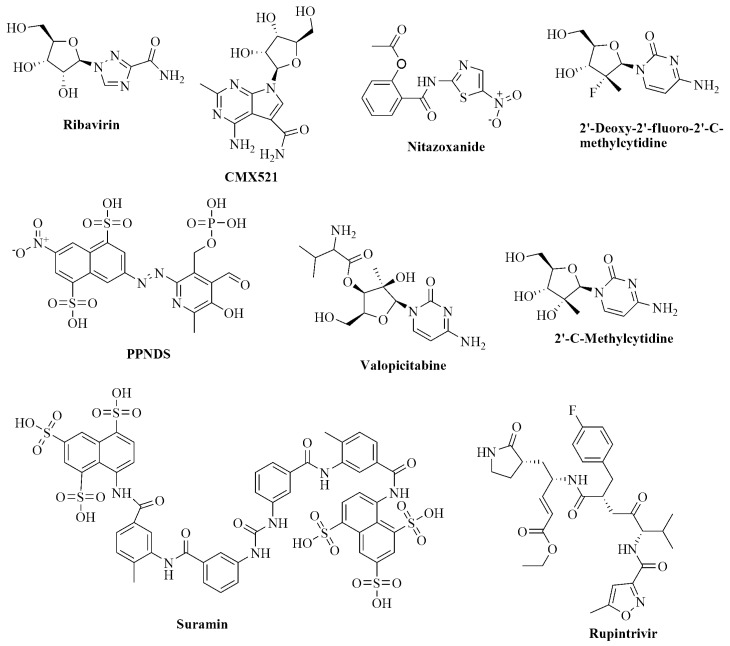
Chemical structure of known nucleoside and non-nucleoside inhibitors of norovirus.

**Figure 2:**
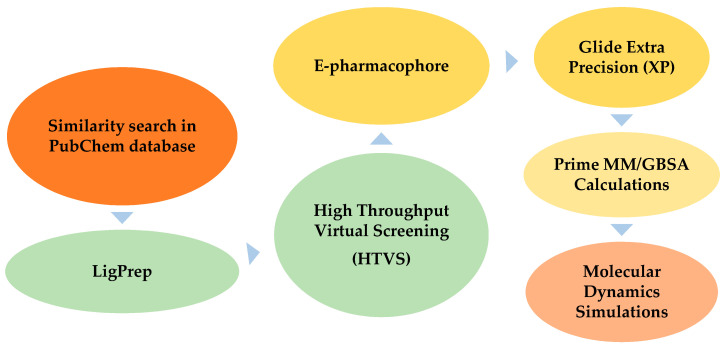
The flowchart of the virtual screening process.

There is a significant demand for human norovirus agents, which can curtail the therapy period, displaying excellent cell membrane permeability and minimal or no side effects. CMX521, a derivative of sangivamycin with the IUPAC name 4-amino-7-[(2*R*,3*R*,4*S*,5*R*)-3,4-dihydroxy-5-(hydroxymethyl)oxolan-2-yl]-2-methylpyrrolo[2,3-d]pyrimidine-5-carboxamide is a nucleoside analog of ribofuranose and derivative. Besides, CMX521 is a drug candidate active against multiple norovirus genotypes, which potently suppresses murine norovirus (MNV) in mice [25]. Interestingly, CMX521 is the first nucleoside analog that has moved to clinical trials (phase 1) to prevent and treat human norovirus [25]. Additionally, the non-nucleoside drug nitazoxanide has proven efficacy against norovirus infection in clinical trials (Figure 1). However, the precise mechanism of nitazoxanide action against norovirus has remained an elusive target for norovirus researchers [26,27,28]. Many library compounds are still similar to CMX521 available in chemical databases, less explored, and worth considering for further studies. We used high throughput virtual screening (HTVS), combined with e-pharmacophore screening and molecular dynamics studies, to efficiently and inexpensively search for new lead compounds that could potentially combat the human norovirus. The flow chart of the methods used is shown in Figure 2. These methodologies have underlined compounds that are structurally similar to the CMX521 molecule, which can be lead to drug design and development against the HuNoV.

## 2. Results and Discussion

CMX521, a nucleoside analog, which suppresses murine norovirus and is presently undergoing clinical trials (phase 1) for treatment and prevention of human norovirus, was employed for a similarity search in a ligand-based VS study. We used the online free database of PubChem, and this resulted in 26,682 structurally similar compounds. The LigPrep panel is used to set up and start ligand preparation calculations. The LigPrep takes 2D or 3D structures and produces the conforming low-energy 3D structures for further use by other programs such as Glide and QikProp. The input and output can be in a structure data file (SDF) or Maestro format. Ligand preparation was done at cellular pH value (7.0 ± 2.0), and probable ionization states were well-considered. The result was 33,560 from 26,682 compounds. The compounds were docked into the active site of the MNV RdRp with the generated receptor grid, using the high throughput virtual screening (HTVS) option in glide’s precision mode, which can sort the output compounds based on docking scores. The docking score of the CMX521 compound (*G*= −6.918) was assigned as the cutoff; meanwhile, the docking score ≥ −6.900 was ranked as active compounds and <−6.900 inactive compounds. A total of 706 compounds with docking scores over −6.900 were the possible MNV RdRp inhibitory molecules and, thus, further passed through e-pharmacophore based screening.

The generation of an e-pharmacophore hypothesis protocol requires a glide pose viewer file of the protein-ligand complex as input [29,30]. With the receptor grid and XP option in the precision mode, the crystal ligand was docked into the MNV RdRp receptor’s active site. The re-docked complex was used further as an input for the e-pharmacophore to create a hypothesis.

**Figure 3:**
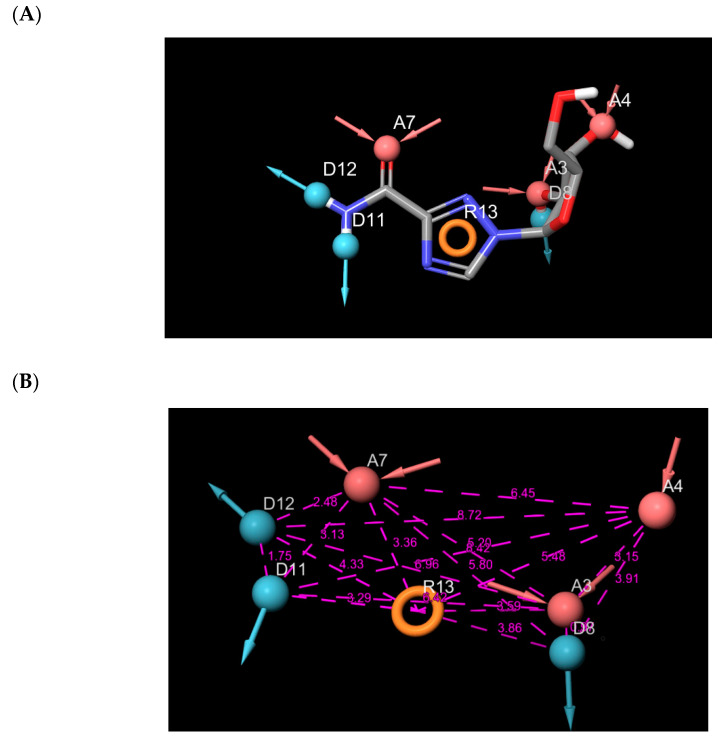
(**A**) Pharmacophore sites generated from the protein data bank structure of murine norovirus RNA-dependent RNA polymerase (MNV-RdRp); (**B**) Distances between the seven pharmacophore sites.

The hypothesis was calculated by enacting Glide XP energy properties over pharmacophore sites based on the protein and ligand underlying data. Seven pharmacophore site hypotheses were engendered from auto and manual pharmacophore settings (Figure 3). Based on the fitness score, 18 hits that resulted from the pharmacophore screening were employed for further studies (Table 1). The fitness score calculation shows firmly how the molecules fitted into the binding pocket using the pharmacophore sites.

**Figure 4:**
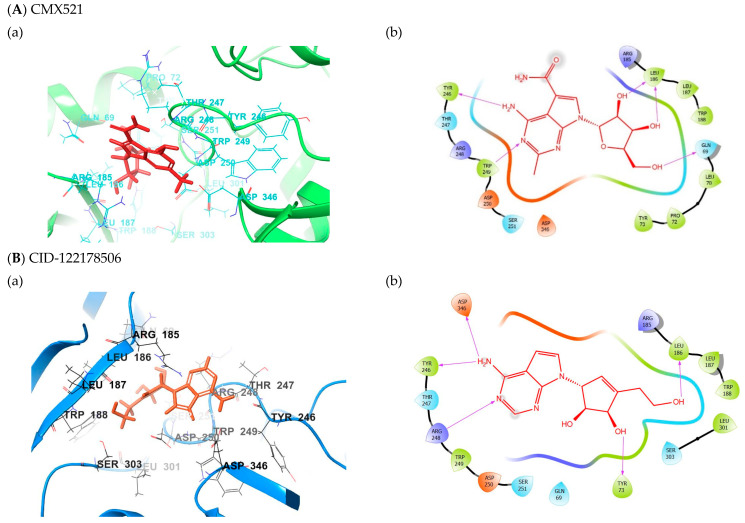

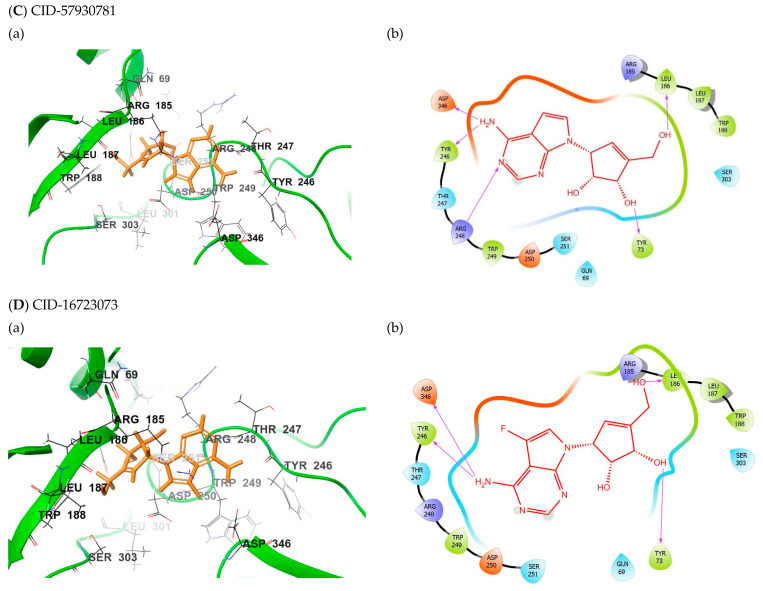

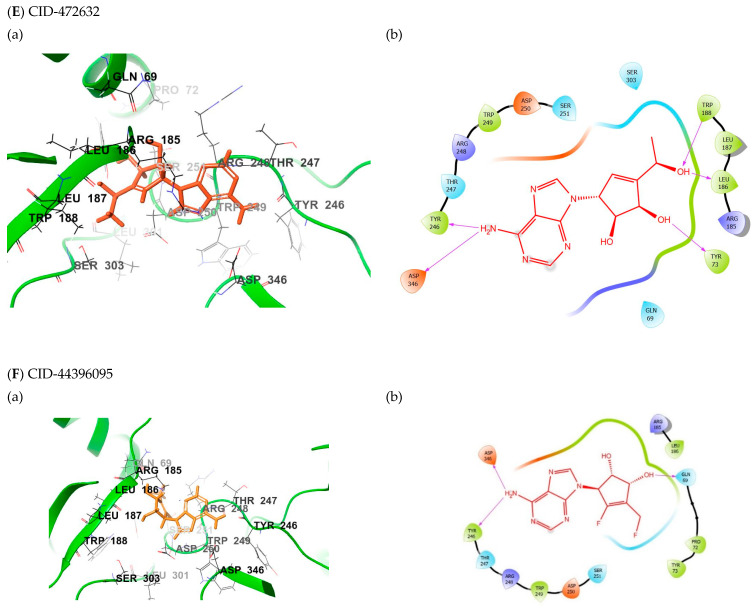

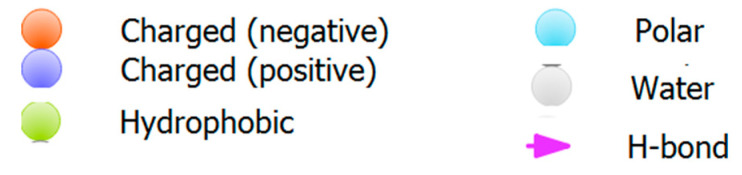
(**a**) The binding mode of the potent compounds as found by XP docking in the active site of MNV RdRp (cartoon) (**A**) CMX521 (**B**) CID-122178506 (**C**) CID-57930781 (**D**) CID-16723073 (**E**) CID-472632 and (**F**) CID-44396095. (**b**) The ligplot of the docked compounds; (**A**) CMX521 (**B**) CID-122178506 (**C**) CID-57930781 (**D**) CID-16723073 (**E**) CID-472632 and (**F**) CID-44396095.

Extra precision (XP) docking was employed to analyze favorable interactions between the ligands and protein. Five compounds top-ranked hits in the binding site of MNV RdRp were explored further for their binding energy calculation using Prime MM-GBSA. The results from the glide XP and Prime MM-GBSA are shown in Table 1. The interactions of the five hits in the binding pocket of MNV RdRp are detailed below (Figure 4). The glide score was used to evaluate the docking of the docked ligands.

The screened ligands were further subject to Lipinski’s five (RO5) rule and reactive pre-filters to eliminate less appropriate compounds in the LigFilter option. The rule states that a drug-like molecule does not have more than five hydrogen bond donors and not more than ten hydrogen bond acceptors. Molecular weights of not more than 500 g/mol and the calculated LogP (clogP) must not be greater than 5 (or mlogP > 4.15/), as compounds that fulfill this optimum prerequisite are considered drug-like [31]. Moreover, the prediction of pharmacokinetic properties also gives insight into the absorption, distribution, metabolism, excretion, and toxicity (ADMET) properties of compounds in the human body before proceeding to the experimental processes. QikProp generates physically relevant descriptors and uses them to perform ADMET predictions [32]. Thus, QikProp was employed for the prediction of ADMET; the parameters considered were: log *K_HSA_* - logarithm of predicted binding constant to human serum albumin (−1.5–1.5); QPlogPw - water/gas partition (4.0 - 45.0); QPlogPC16 –hexadecane/gas partition (4.0–18); log BB - logarithm of predicted blood/brain barrier partition coefficient (−3.0–1.2); Caco-2 - cell membrane permeability (<25 poor >500 good); QP*_polrz_* - predicted polarizability (13–70); log *HERG* - the predicted IC_50_ value for the blockage of HERG K^+^ channels (concern below −5); QPPMDCK - predicted MDCK cell permeability in nm/sec (<25 poor >500 great); log *K*_p_ - predicted skin permeability and 95% of drugs: (−8–−1); log *K_HSA_* - logarithm of predicted binding constant to human serum albumin (−1.5–1.5), Human Oral Absorption (HOR), 1–low, 2–medium, 3–high. All the hit compounds fulfilled the optimum requirement for drug-like molecules. The results from the QikProp are detailed in Table 2 and Table 3.

### 2.1. Binding Mode of the CMX521 and Ribavirin—MNV RdRp Complex

The reference compound CMX521 has the lowest docking score (XP glide score) of −6.918 kcal/mol. The ligand fitted nicely into the binding pocket consisting of residues Try246, Thr247, Arg248, Trp249, Asp250, Ser251, Asp346, Arg185, Leu186, Leu187, Gln69, Leu70, Tyr73, Pro72 and Trp188 (Figure 4). This molecule’s putative binding mode in the MNV RdRp active site indicated that H-bonds (hydrogen bonds) and hydrophobic interactions mainly drove the protein-ligand binding site. In this regard, the nitrogen and hydrogen atom of the amine functional group of the 2-methylpyrrolopyrimidine-5-carboxamide ring formed H-bond interactions with the hydrogen atom of the amino group of Trp249 (bond distance = 2.70 Å) and the carbonyl oxygen of Tyr246 (bond distance = 2.44 Å). Meanwhile, the hydrogen atoms of the hydroxyl group on position 1 and 2 of ribonucleoside formed H-bonds interaction with carboxyl oxygen of Leu186 (bond distance = 2.41 and 2.12 Å), and the oxygen atom of the hydroxyl in the position formed H-bond with the hydrogen atom of the amine group of Leu186. The hydroxyl substituent at positions 1 and 2 of ribonucleoside moiety (Ribavirin) established H-bonds with Aps250 and Thr309, whereas the hydroxyl methyl interacted with Tyr344 and Asp346. It was also observed in the docked complex of CMX521, amino acid, Ser251 established H-bonds interaction with the oxygen atom of 4-hydroxyl (ribonucleoside ring) through its carboxyl oxygen and the amine group (hydrogen atom). In contrast, the methylene group’s of the hydrogen atom formed H-bond interaction with the hydroxyl group of Gln69. Further, the methyl and pyrrole group of CMX521 molecule formed hydrophobic interaction with side chain Trp249, Arg248 and, Arg185 (Figure 4).

### 2.2. Binding Mode of the CID-122178506—MNV RdRp Complex

The detailed visual view of CID-122178506 indicates that the two hydrogen atoms of the amine group present in pyridopyrimidine interact with the carboxyl oxygen atom of Asp346 and the hydroxyl oxygen atom of Tyr246 (bond distance = 2.06 and 2.31 Å) (Figure 4). The pyrrole and pyrimidine interact with sidechain Arg185 and Arg248 via hydrophobic (π-alkyl) and electrostatic (π-cation). The two hydrogen atoms in the 2-hydroxyethyl group singly interact with the carboxyl oxygen of Asp250 and Ser303, whereas the amino acid Try73 was squeezed between the methine and hydroxyl proton in position one of the cyclopentene-1,2-diol ring (bond distance = 1.89 and 2.89 Å) to established H-bonding. Meanwhile, the hydrogen and oxygen in the primary hydroxyl group follow suit to interact separately with the hydroxyl oxygen atom of Leu186 and the hydrogen atom of the amine group of Trp188.

### 2.3. Binding Mode of the CID-57930781—MNV RdRp Complex

The ligand-binding landscape of CID-57930781—MNV RdRp complex is similar to the compound CID-122178506 (−7.587 kcal/mol). The nitrogen atom and the amine (hydrogen atom) functional group of the pyridopyrimidine ring formed H-bond interactions with the amino group of Trp249 (bond distance = 2.45 Å) and the carbonyl oxygen of Tyr246 (bond distance = 2.56 Å), which is also observed in the binding mode of the reference molecule. However, the sidechain of amino acids Arg185 and 248 established electrostatic (π-cation) and hydrophobic (π-alkyl) interaction with pyrrole moiety. Interestingly, the cyclopentene-1,2-diol ring hydrophobically interacts with the sidechain of Arg185; the methine proton in position one of cyclopentene-1,2-diol, H-bonded with the carbonyl oxygen atom of Gln69 (bond distance = 2.80 Å). The methine and hydroxyl proton in position two of cyclopentene-1,2-diol interact with the hydroxyl group of Tyr73 with bond distances of 1.78 and 3.03 Å. The methylene group’s hydrogen atoms also H-bonded with the carboxyl oxygen of Asp250; the amino acids Leu186 and Trp188 interact in the same form as observed in the binding mode of CID-122178506 (Figure 4).

### 2.4. Binding Mode of the CID-16723073—MNV RdRp Complex

The calculated glide score of CID-16723073 was -6.910 kcal/mol (Table 1) and displayed low binding energy compared to CID-122178506 and CID57930781 (Δ*G^bind^* = −53.900 kcal/mol) (Table 1). The presence of a fluorine atom on the pyrrole ring led to the decreased binding energy of CID-16723073. Surprisingly, the ligand packed tightly into the binding site and made some significant interactions, including halogen bonding. The fluorine atom formed halogen bonds with the carboxyl oxygen atom of Asp346 and Asp250. The pyridopyrimidine ring, as well as the nitrogen atom in the pyrimidine ring in ligand CID-16723073, interacted in the same form as observed in the compound CID-122178506. Notably, the amine functional group interacts with the carboxyl oxygen atom of Asp346 and the hydroxyl oxygen atom of Tyr246 (bond distance = 2.33 and 2.27 Å). The nitrogen atom and the pyrrole pyrimidine ring interact with the sidechain of Arg248 and Arg185 via hydrophobic (π-alkyl) and electrostatic (π-cation). Moreover, the cyclopentene-1,2-diol moiety oriented towards the binding pocket consisting of amino acids Ser251, Tyr73, Leu186, and Trp188. The cyclopentene-1,2-diol hydrophobically interacts with the sidechain of Leu186; the hydrogen atom of the alkyl hydroxyl was sandwiched between the hydrogen atom of the amine group in the indole moiety on Trp188 (2.67 Å) and the hydroxyl oxygen of Leu186 (1.72 Å). The hydroxyl oxygen atoms in positions one and two of cyclopentene-1,2-diol interact with the hydroxyl group (hydrogen atom) of Ser251, the amino acid Tyr73 H-bonded with the hydrogen atom of methylene group attached to cyclopentene-1,2-diol. Figure 4.

### 2.5. Binding Mode of the CID-472632—MNV RdRp Complex

Compound CID-472632 (Δ*G^bind^* = −59.285 kcal/mol) is a derivative of pyrimidine-imidazole; the detailed binding interaction shows that the amine functional group’s two hydrogens on the pyrimidine-imidazole ring formed H-bonds with carboxyl oxygen of Asp346 (2.04 Å) and hydroxyl oxygen of Tyr246 (1.86 Å), respectively. The amine group (hydrogen atom) of amino acid Arg248 formed H-bond with the nitrogen atom in the pyrimidine ring of CID-472632. In contrast, the sidechain of Arg248 hydrophobically interacts with the pyrimidine moiety. Further, hydroxyl protons attached to the cyclopentene-1,2-diol ring interact with the hydroxyl group of Tyr73 (1.78 Å) and amino acid Gln69 (2.80 Å), respectively. Also, the methyl group formed hydrophobic interaction with the side chain of Trp188 (Figure 4).

### 2.6. Binding Mode of the CID-44396095—MNV RdRp Complex

Although CID-472632 and CID-44396095 are derivatives of pyrimidine-imidazole, the ligand-binding landscape of the CID-44396095-MNV RdRp complex (Δ*G^bind^* = −61.969 kcal/mol) is different from its counterpart (CID-472632 docked complex). The visual interaction of CID-44396095 in the active pocket of MNV-RdRp shows that the amine functional group’s two hydrogens on the pyrimidine-imidazole ring moved closer to amino acids Asp346 and Tyr246 to formed H-bonds with carboxyl oxygen of Asp346 (1.90 Å) and hydroxyl oxygen of Tyr246 (1.89 Å), respectively. Interestingly, the amine group (hydrogen atom) of Asp250 H-bonded with the imidazole ring of CID-44396095. Whereas the pyrimidine moiety hydrophobically interacts with the Arg248 sidechain. Remarkably, the fluorine substituent at position four of cyclopentene-1,2-diol ring established three H-bonds interaction with amino acid Ser251; the fluoro atom interacted with amine (hydrogen atom, 2.11 Å), methylene (hydrogen atom, 2.18 Å), and hydroxyl (hydrogen atom, 1.89 Å) groups of Ser251. At the same time, it formed an H-bond (bond distance = 2.90 Å). The fluoro atom of fluoromethyl attaching to cyclopentene-1,2-diol ring at position three follows suit to form three H-bonds with Ser251; the fluoro atom interacts with methylene group of Ser251 and Arg248 (hydrogen atom, 2.67, 3.00 Å) and the hydroxyl group (hydrogen atom, 1.87 Å) of Ser251. In contrast, the methyl group hydrophobically interacts with the sidechain of Arg248 and pyrrolidine ring of Pro72. Further, the 2-OH also contributes to the complex’s stability by interacting with the carbonyl oxygen of Gln69 via H-bond (Figure 4).

Furthermore, molecular dynamics simulations (MDSs) of 50 ns was performed to appraise conformational variations in the ligand-binding domain (LBD) of CMX521 and the lead compounds. Meanwhile, only a single MDSs was achieved; the possible limitation is that the system may not reach equilibrium. It is tricky to assess the ligand-binding mode’s stability only from a single simulation when it does not reach equilibrium; thus, the ligand’s binding mode is deemed erratic during the MD simulation. Interestingly, the root mean square deviation (RMSD) results showed how the complexes’ MD simulations reached equilibrium before 50 ns for single MDSs, consequently showing the ligand-binding mode’s proportional stability.

**Figure 5:**
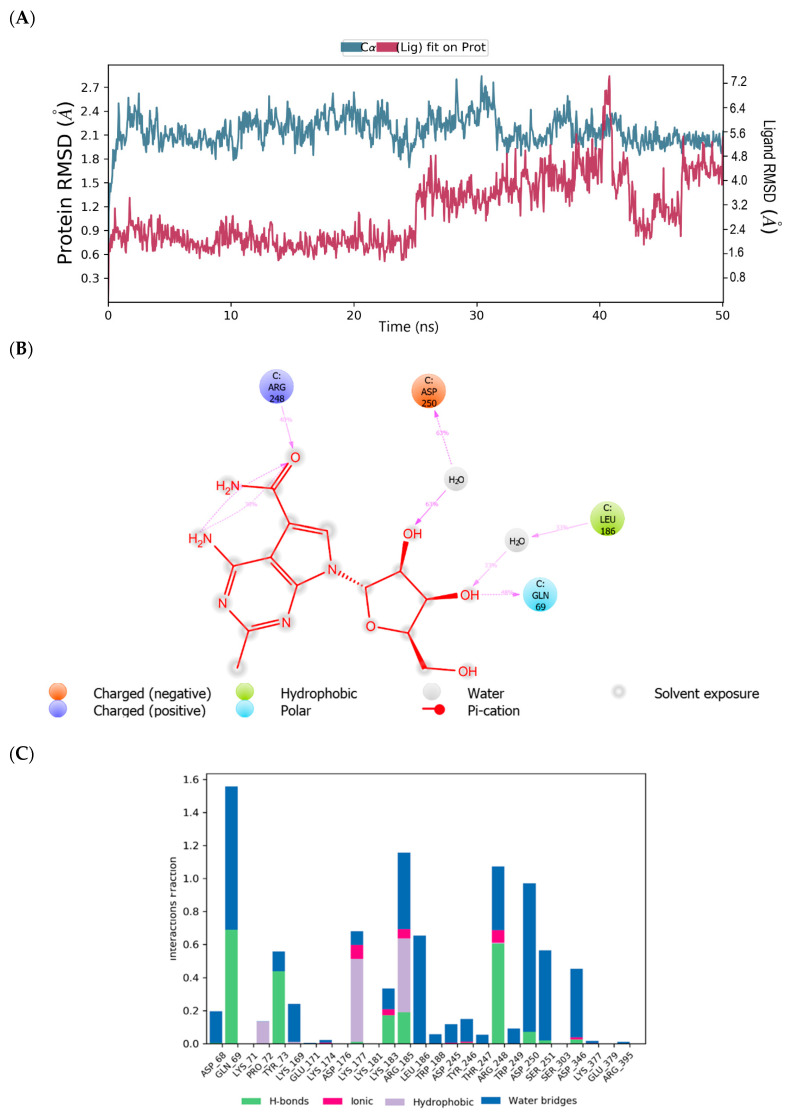

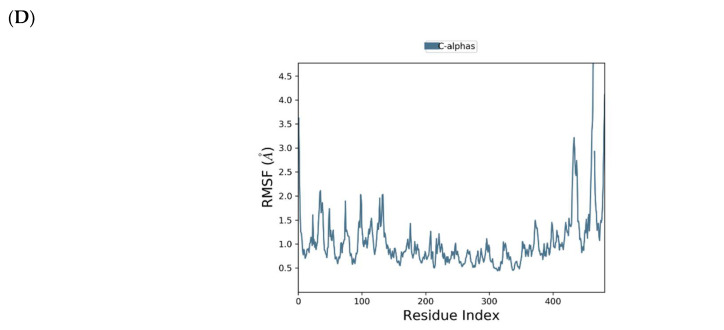
(**A**) MDSs trajectory output revealing the root-mean-square deviation (RMSD) plot of CMX521 bound to MNV RdRp; (**B**) Binding interaction of CMX521-MNVRdRp after MDSs; (**C**) Ligplot representing interaction of CMX521 with different residues of MNV RdRp throughout the simulation trajectory (**D**) Graphical depiction of root-mean-square fluctuation (RMSF) of CMX521 with different residues of MNV RdRp.

**Figure 6:**
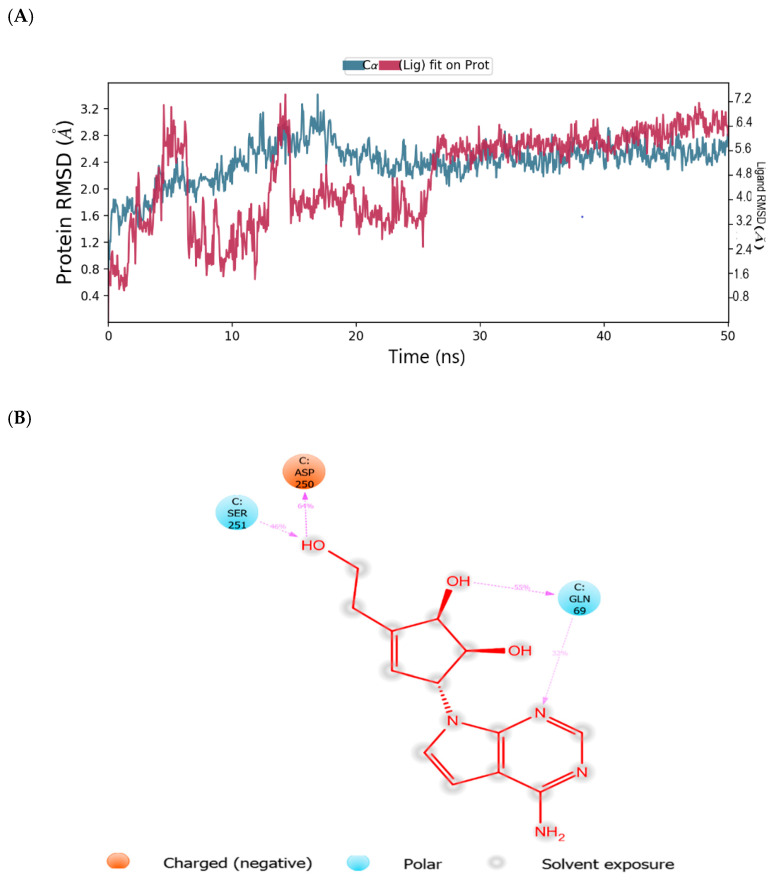

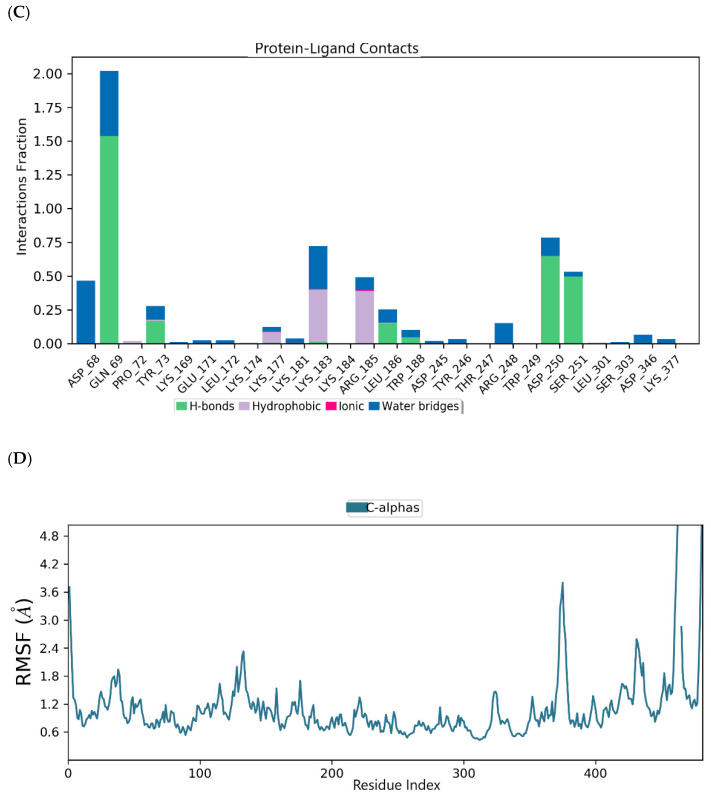
(**A**) MDSs trajectory output revealing the root-mean-square deviation (RMSD) plot of CID-122178506 bound to MNV RdRp; (**B**) Binding interaction of CID-122178506 -MNV RdRp after MDSs; (**C**) Ligplot representing interaction of CID-122178506 with different residues of MNV RdRp throughout the simulation trajectory (**D**) Graphical depiction of root-mean-square fluctuation (RMSF) of CID-122178506 with different residues of MNV RdRp.

**Figure 7:**
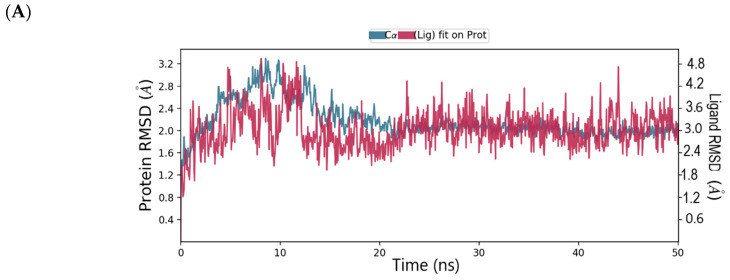

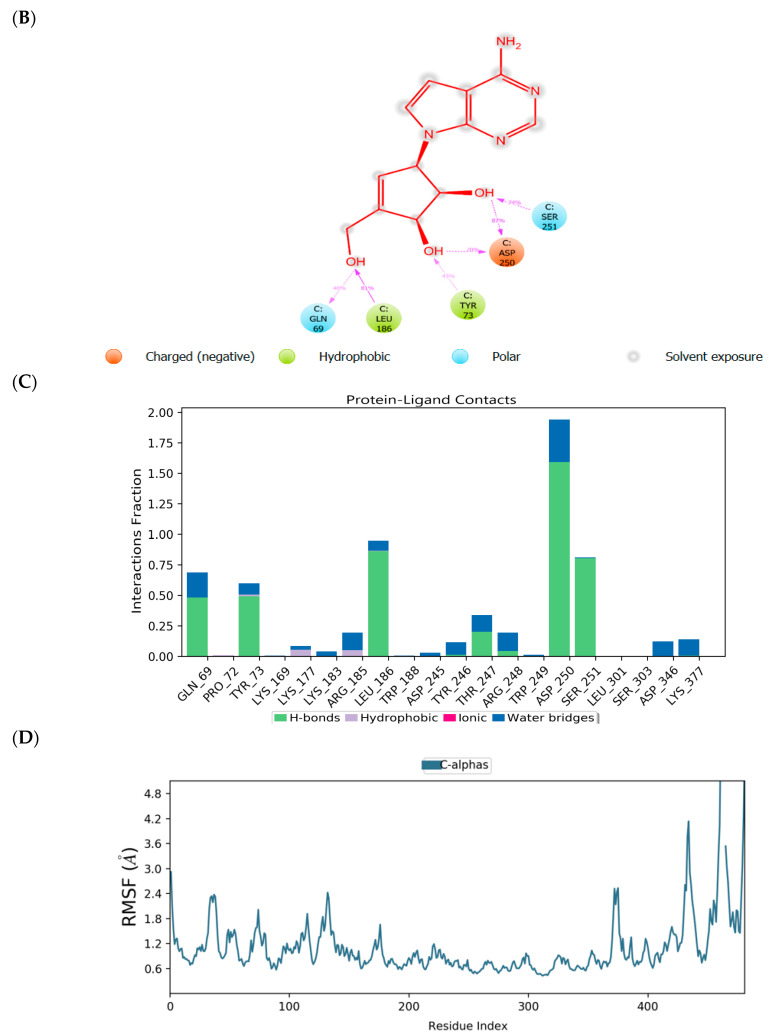
(**A**) MDSs trajectory output revealing the root-mean-square deviation (RMSD) plot of CID-57930781 bound to MNV RdRp; (**B**) Binding interaction of CID-57930781 -MNV RdRp after MDSs; (**C**) Ligplot representing interaction of CID-57930781 with different residues of MNV RdRp throughout the simulation trajectory (**D**) Graphical depiction of root-mean-square fluctuation (RMSF) of CID-57930781 with different residues of MNV RdRp.

**Figure 8:**
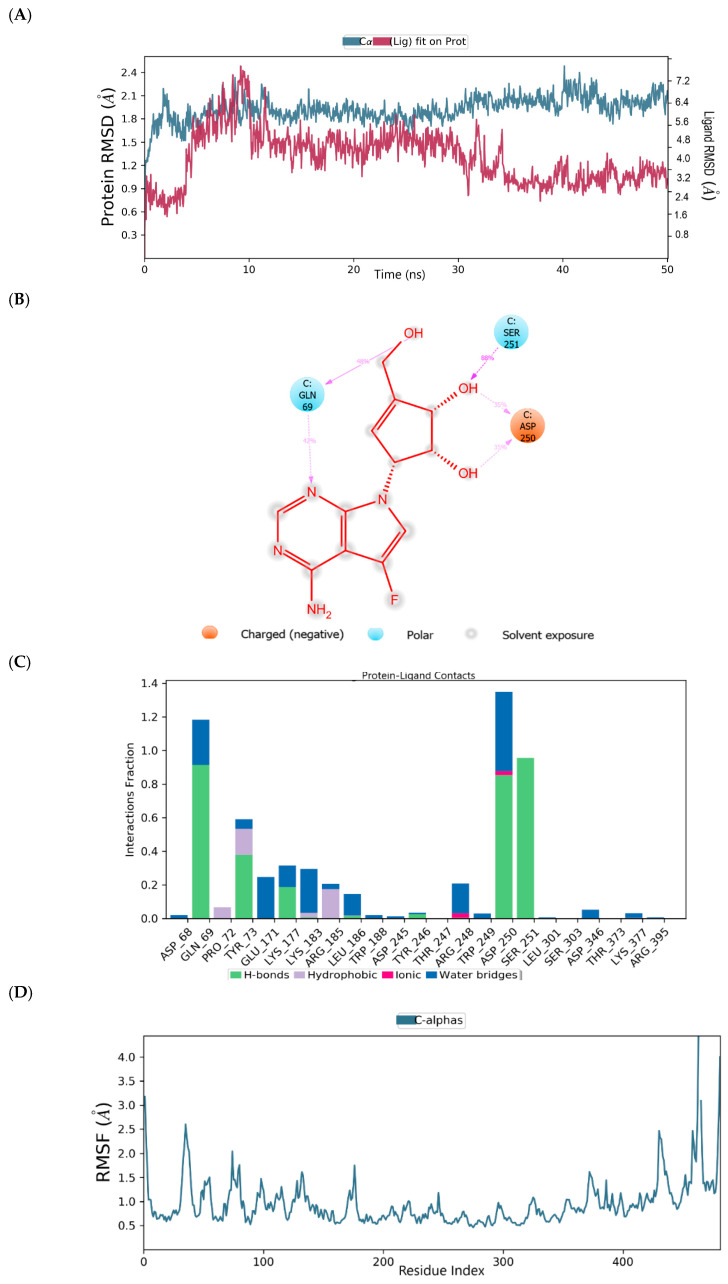
(**A**) MDSs trajectory output revealing the root-mean-square deviation (RMSD) plot of CID-16723073 bound to MNV RdRp; (**B**) Binding interaction of CID-16723073 -MNV RdRp after MDSs; (**C**) Ligplot representing interaction of CID-16723073 with different residues of MNV RdRp throughout the simulation trajectory (**D**) Graphical depiction of root-mean-square fluctuation (RMSF) of CID-16723073 with different residues of MNV RdRp.

**Figure 9:**
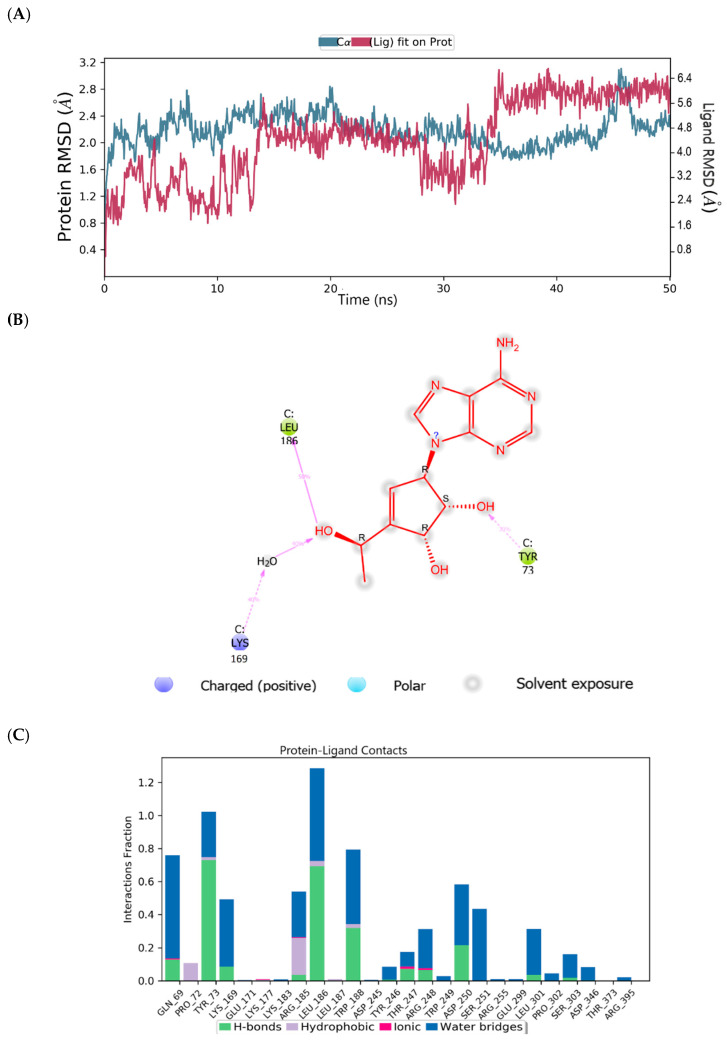

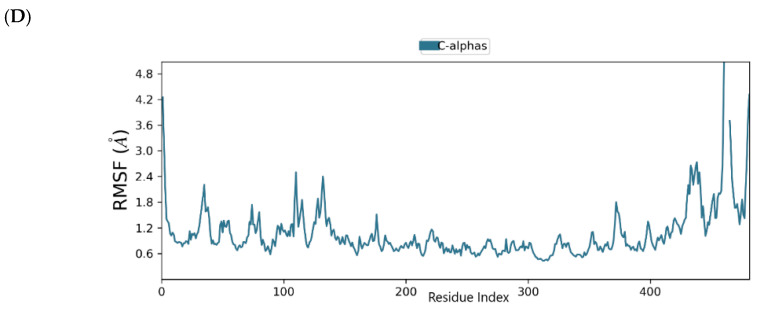
(**A**) MDSs trajectory output revealing the root-mean-square deviation (RMSD) plot of CID- 472632 bound to MNV RdRp; (**B**) Binding interaction of CID- 472632 -MNV RdRp after MDSs; (**C**) Ligplot representing interaction of CID- 472632 with different residues of MNV RdRp throughout the simulation trajectory (**D**) Graphical depiction of root-mean-square fluctuation (RMSF) of CID- 472632 with different residues of MNV RdRp.

**Figure 10:**
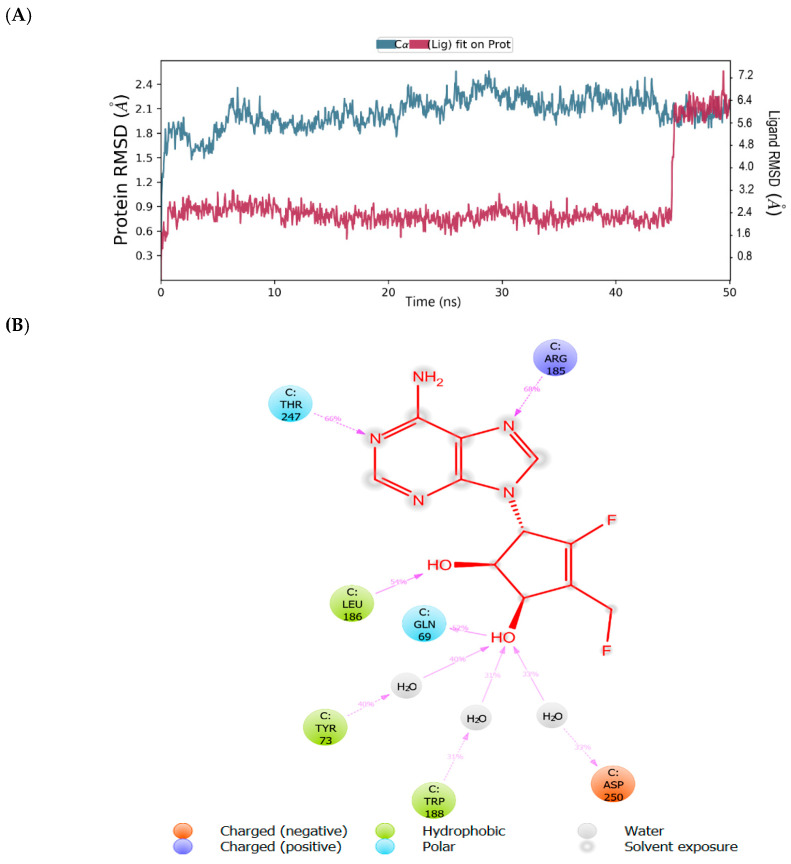

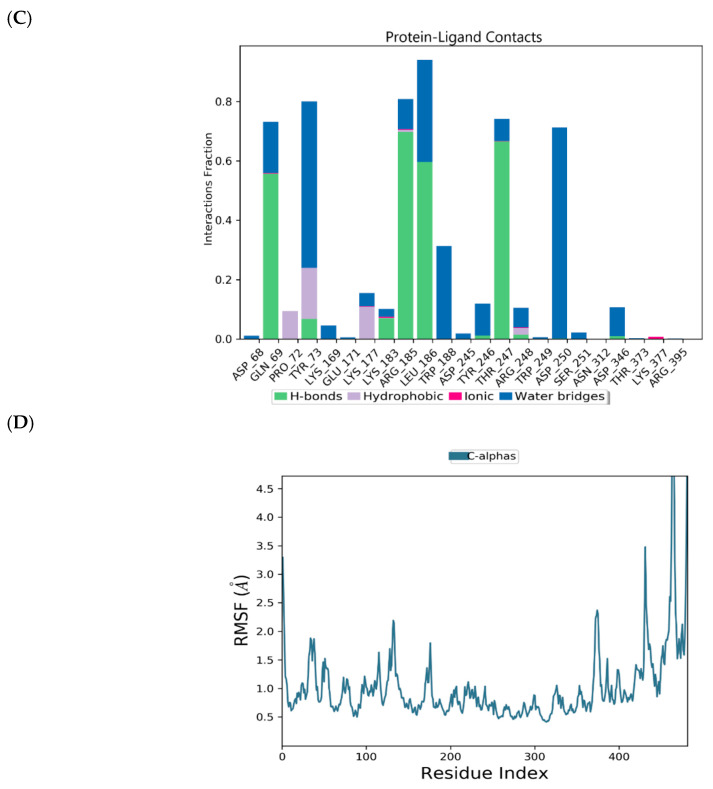
(**A**) MDSs trajectory output revealing the root-mean-square deviation (RMSD) plot of CID-44396095 bound to MNV RdRp; (**B**) Binding interaction of CID-44396095 -MNV RdRp after MDSs; (**C**) Ligplot representing interaction of CID-44396095 with different residues of MNV RdRp throughout the simulation trajectory (**D**) Graphical depiction of root-mean-square fluctuation (RMSF) of CID-44396095 with different residues of MNV RdRp.

The root mean square deviation (RMSD) and root mean square fluctuation (RMSF) plots were used to examine the binding affinity between ligands and the MNV-RdRp active site. The RMSD of the reference compound CMX521 in the binding pocket of MNV-RdRp during the 50 ns simulation showed nonconformities between 1.2 and 5.4 Å (Figure 5a). After the 50 ns simulations, the LBD of CMX521 showed that the hydroxyl group at position one and two had formed H-bonds with Asp250 and Leu186 through water molecules; the amino group established 48% H-bond interaction with Arg248; and the 2-hydroxyl H-bonded with Gln69 (Figure 5b). The RMSD of MNV-RdRp was between 1.2 and 2.7Å, which depicted the docked complex’s stability during the simulation. The RMSD, protein-ligand contact, interactions and RMSF of CMX521 after MDS are shown in Figure 5a–d. The MDSs analysis of the five lead compounds was also examined, and there were vast differences in the analyzed results. The RMSD, protein-ligand contact, interactions, and RMSF of CID-122178506, CID-57930781, CID-16723073, CID-472632, and CID-44396095 after MDS are detailed in Figure 6a–d, Figure 7a–d, Figure 8a–d, Figure 9a–d and Figure 10a–d. Besides, the difference in the RMSD of the protein-ligand docked complex of lead compounds (Figure 6a, Figure 7a, Figure 8a, Figure 9a and Figure 10a) leads to H-bonds’ variability association between the complexes. The MDSs analysis visualization revealed that the hit compound CID-57930781 (Figure 7a–d), a pyridopyrimidine nucleoside analog, and CID-44396095 (Figure 10a–d), a pyrimidine-imidazole derivative, were found to produce better results when compared to other compounds; both compounds satisfied the Lipinski’s rule of five compared to CMX521. Surprisingly the 1,2-hydroxyl and primary hydroxyl of the cyclopentene-1,2-diol ring (CID-57930781) maintained their interactions with Gln69, Leu186, Tyr73, Asp250, and Ser251, in the absence of water bridges and show superior interaction compare to CID-122178506, CID-16723073, and CID-472632 (Figure 6b, Figure 8b and Figure 9b). Interestingly, the 1,2-dihydroxyl substituents on the cyclopentene-1,2-diol ring of CID-44396095 maintained their interactions with Leu186, Gln69, and also with amino acid Trp188, Tyr73, and Asp250 through water viaduct (Figure 10b). The RMSD of CID-57930781 and CID-44396095 indicates excellent stability in the binding site of MNV-RdRp (Figure 7a and Figure 10a), suggesting these molecules may epitomize potential inhibitors. They might also reduce HNV infectious disease’s hitches by acting on RdRp, in vitro, and in vivo anti-norovirus activity. H-bonds are imperative interactions in the stability of protein−ligand complexes. Besides, they perform a crucial role in ligand binding due to their strong influence on drug specificity, metabolization, and adsorption. The interaction of CID-57930781 and CID-44396095 with the critical amino acid was unswerving through the 50 ns MDSs, via H-bond contact, as shown in Figure 7c and Figure 10c compare to Figure 6c, Figure 8c and Figure 9c). Accordingly, the peaks in the RMSF plot indicate the most mutable residues of the target protein. High fluctuations in the residues were observed at the beginning and end of the plots; most residues’ fluctuations were less than 2 Å (Figure 5d, Figure 6d, Figure 7d, Figure 8d, Figure 9d and Figure 10d). 

## 3. Material and Methods

### 3.1. Data Software and Visualization

In this study, we utilized a molecular modeling package from Schrödinger’s Drug Discovery Suite 2019-3 (Schrödinger, Inc., LLC, New York, NY, USA) and Discovery Studio *Visualizer* software developed by Accelrys (*Dassault Systemes*, *BIOVIA* Corp., *San Diego*, CA, USA). Maestro provided entree to all Schrödinger modules alongside expertise to organize and evaluate data as a portal interface of Schrödinger.

### 3.2. Dataset and Compound Library Preparation

In the VS workflow’s initial step, the CMX521 molecule was used as a prototype for a similarity search in the PubChem online database with a 70% threshold. PubChem, a free web-based server, currently (July 2020) contains ~267 million substances (SID), 103 million unique chemical structures (CID), and ~ 1 million bioassays (AID) and is supported by the US National Library of Medicine [33]. These millions of compound records and bioassay data pools are excellent for drug discovery and development [34]. Our similarity search of the CMX521 molecule resulted in a library of 26,682 compounds, downloaded as Structure-Data File (SDF) format for further analysis.

### 3.3. Ligand Preparation

The ligands were obtained from the Pubmed database. Further, the ligands were subjected to ligand preparation using the Ligprep module of Schrodinger Suite. During the process, there was a conversion of 2D to 3D structures, the OPLS (optimized potentials for liquid simulations) all-atom 2005 force field was used for optimization. Which generates the lowest possible energy conformation of the ligand structures, tautomeric states and, ionization on a cellular pH value (7.0 ± 2.0) using the Epik tool [35,36,37].

### 3.4. Receptor Preparation and Grid Generation

MNV RdRp and HuNoV are categorized under the norovirus genus of the *Caliciviridae* family hence share many biological and genomic features. It is worth saying that MNV RdRp serves as a proxy to expound the mechanisms of infection and replication of HuNoV because MNV RdRp is the only norovirus that imitates in the cell culture system and small animal models [38,39,40]. RNA-dependent RNA polymerase (RdRp) is one of the multipurpose *enzymes* of the RNA *viruses* that is vital for replication and transcription of the viral *genome* using ribonucleotide triphosphates as substrates, hence making this virally encoded enzyme one of the critical targets for developing novel antiviral agents [41,42]. In the context of MNV RdRp, the crystal structure of murine norovirus RdRp with its native ligand ribavirin was retrieved from the protein data bank (PDB; 3FSU) with a resolution of 2.5Å and imported into the protein preparation wizard in the Schrodinger suite 2019-3 [43]. In the first phase of protein preparation, the MNV RdRp was preprocessed by assigning bond orders, adding hydrogen atoms to the structure, and correcting metal ionization states to ensure proper formal charge and force field treatment. Simultaneously with the removal of crystallographic water molecules beyond 5 Å, the creation of disulfide bonds, cap protein termini with ACE (N-*acetyl*) and *NMA* (N-methyl amide) *groups*, and the generation of heteroatoms was done using the Epik tool. The second phase involved reviewing and modifying the protein; the co-crystal ligand, together with chain A and B of the protein, was removed. Finally, optimization of the protein’s hydrogen bond and restrained minimization was performed. The Glide module’s receptor grid generation route (Grid-based Ligand Docking with Energetic) implemented in the Schrodinger suite was utilized to generate a receptor grid file for the prepared MNV RdRp protein [44].

### 3.5. Structure-Based Virtual Screening

#### 3.5.1. High Throughput Virtual Screening

Further, Glide protocol, which includes high throughput virtual screening (HTVS), was used to performed docking of the compounds downloaded into the active site of MNV RdRp [44,45]. In the HTVS mode, the compounds with bad hits are quicker to eliminate. The glide XP mode utilized a more complex scoring function than the HTVS and SP glide score. This step eliminates false positives that pass through the glide with standard precision; moreover, glide with the extra-precision mode (XP) can penalize ligands that are not suitable for the receptor conformation.

#### 3.5.2. E-Pharmacophore Hypothesis Generation and Database Screening

The crystal-ligand of MNV RdRp was docked into the receptor’s active site. The root mean square deviation (RMSD) was obtained from the docked structure’s superposition, and the crystal structure was evaluated. Docking into the binding site defined by MNV RdRp was performed using glide with the extra-precision mode (XP). The protein-ligand complexes thus served as an input for generating pharmacophore sites. The structured e-pharmacophore was used as a probe for virtual screening. Phase module of Schrodinger suite was utilized for pharmacophore generation using a set of three chemical features: aromatic ring, H-bond donors, and H-bond acceptors, as shown in Figure 1 [46,47]. Database hits were ranked based on this fitness score. The ligands with the best fitness score were docked with glide XP into the MNV RdRp receptor’s active site.

#### 3.5.3. Extra Precision Docking (XP)

Extra precision (XP) from glide protocol was employed for docking the hits into the active site of MNV RdRp [45]. The glide XP mode utilized a more complex scoring function. In this step, glide XP can penalize ligands that are not suitable for the receptor conformation. Finally, five-hit compounds were selected from this phase. The docked complexes are analyzed, visually examined, and the five compounds’ results with their corresponding glide score are presented in Table 1. The five compounds’ free binding energy was examined using the Prime MM-GBSA module Schrodinger suite [48].

### 3.6. Molecular Dynamics Simulations

The lead compounds and the reference compound were explored for their thermodynamic behavior and the binding mode’s stability in the active pocket of MNV-RdRp using molecular dynamics simulations (MDSs) [49,50]. Desmond module in the Schrodinger suite was used to perform the MDSs of all the docked compounds [51]. The protein-ligand complexes were first; prepared using protein preparation workflow, followed by system model building. The system builder option was used to integrate the simple point charge (SPC); the water model was incorporated in the docked protein-ligand complex in an orthorhombic periodic boundary of the box volume. Neutralization of the system was performed by adding one chlorine ion depending on the system’s total charge, and a salt concentration of 0.15 M was added. MDS studies were also carried out with a periodic boundary condition in the number of atoms, pressure, temperature (NPT) ensemble, the temperature at 300 K, one atmospheric pressure, and finally relaxed using the default relaxation protocol. Finally, complexes succumbed to a production run of 50 ns. The simulation job was performed throughout 50 ns. Root mean square deviation (RMSD) and root mean square fluctuation (RMSF) of the complexes were analyzed using depicted simulation-interaction diagrams.

## 4. Conclusions

This study employed a similarity search of CMX521 followed by HTVS with e-pharmacophore screening, XP docking, and molecular dynamics simulations. These resulted in the prediction of two potent inhibitors, CID-57930781 and CID-44396095. Moreover, the ADMET prediction revealed that the two compounds achieved optimum therapeutic efficacy. The two ligands fitted into the binding pocket of the MNV RdRp. Based on the molecular docking results, ADMET, and molecular dynamics calculations, compound CID-57930781 and CID-44396095 appear as a new structural ground to be exploited for the design and development as a new HuNov agent. A step further in the lead compound’s experimental investigations will make available specific analogous design indications with an enhanced pharmacological profile.

## Figures and Tables

**Table 1:** The results of the lead compounds from the Glide XP docking and Prime MM-GBSA.

Compound	Glide Score(kcal/mol)	Fitness Score	Prime MM-GBSA (dG bind kcal/mol)
CID-122178506	−7.073	2.356	−62.554
CID-57930781	−7.587	2.336	−62.528
CID-16723073	−6.901	2.326	−53.900
CID-472632	−7.720	2.201	−59.285
CID-44396095	−7.390	2.118	−61.969

**Table 2:** Physicochemical profiles of the lead compounds and the reference compound using Qikprop.

Compound Identifier	MW	SASA	HBA	HBD	PSA	RO5
CID-122178506	276.29	492.310	7.6	5	111.360	0
CID-57930781	262.27	478.545	7.6	5	111.298	0
CID-16723073	280.258	482.612	7.6	5	112.376	0
CID-472632	277.28	489.838	9.1	5	128.212	0
CID-44396095	283.24	484.955	7.4	4	109.498	0
CMX521	323.308	542.358	11.8	7	169.66	1

MW: Molecular weight (g/mol); Total solvent accessible surface area (SASA) (300.0–1000.0 Å); HBA: number of hydrogen bond acceptors (2–20); HBD: number of hydrogen bond donors (0–6); PSA: Polar Surface Area (7–200 Å^2^); RO5 - Lipinski’s rule of five.

**Table 3:** Pharmacokinetic properties of the lead compounds and the reference compound using Qikprop.

Entry	QPpolrz	QPlogPw	QPlogPo/w	QPlogS	QPlogHERG	QPPCaco	QPlogBB	QPlogKp	QPlogKhsa	HOR	QPPMDCK
1	25.008	16.812	−0.252	−2.033	−4.173	78.500	−1.721	−4.552	−0.650	2	31.611
2	24.349	17.097	−0.366	−2.034	−4.319	95.715	−1.591	−4.399	−0.675	2	39.166
3	24.727	16.87	−0.128	−2.264	−4.069	100.678	−1.446	-4.478	−0.636	3	70.187
4	25.164	18.530	−0.953	−2.065	−4.036	39.974	−1.927	−5.314	−0.720	2	15.242
5	25.376	15.542	0.081	−2.815	−4.172	97.571	−1.308	−4.766	−0.549	3	102.922
6	28.005	24.005	−1.897	−2.282	−4.182	14.282	−2.542	−6.261	−0.873	2	5.011

1- CID-122178506; 2- CID-57930781; 3- CID-16723073, 4- CID-472632; 5- CID-44396095; 6- CMX521. log *K_HSA_*: logarithm of predicted binding constant to human serum albumin (−1.5–1.5); QPlogPw: water/gas partition (4.0–45.0); QPlogPC16 –hexadecane/gas partition (4.0–18); log BB: logarithm of predicted blood/brain barrier partition coefficient (−3.0–1.2); Caco-2: cell membrane permeability (<25 poor >500 good); QP*_polrz_*: predicted polarizability (13–70 Å^3^); log *HERG*: the predicted IC_50_ value for the blockage of HERG K^+^ channels (concern below −5); QPPMDCK: predicted MDCK cell permeability in nm/sec (<25 poor >500 great); log *K*_p_: predicted skin permeability and 95% of drugs: (−8–−1); Human Oral Absorption (HOR)- 1-low, 2-medium, 3-high.
